# Vitamin D Mitigates Hepatic Fat Accumulation and Inflammation and Increases SIRT1/AMPK Expression in AML-12 Hepatocytes

**DOI:** 10.3390/molecules29061401

**Published:** 2024-03-21

**Authors:** Eugene Chang

**Affiliations:** Department of Food and Nutrition, Gangneung-Wonju National University, Gangneung-si 25457, Gangwon State, Republic of Korea; echang@gwnu.ac.kr; Tel.: +82-33-640-2338

**Keywords:** fat accumulation, inflammation, non-alcoholic fatty liver disease (NAFLD), NOD-like receptor family pyrin domain-containing 3 (NLRP3), sirtulin 1 (SIRT1), vitamin D

## Abstract

Emerging evidence has demonstrated a strong correlation between vitamin D status and fatty liver disease. Aberrant hepatic fat infiltration contributes to oxidant overproduction, promoting metabolic dysfunction, and inflammatory responses. Vitamin D supplementation might be a good strategy for reducing hepatic lipid accumulation and inflammation in non-alcoholic fatty liver disease and its associated diseases. This study aimed to investigate the role of the most biologically active form of vitamin D, 1,25-dihydroxyvitamin D (1,25(OH)2D), in hepatic fat accumulation and inflammation in palmitic acid (PA)-treated AML-12 hepatocytes. The results indicated that treatment with 1,25(OH)2D significantly decreased triglyceride contents, lipid peroxidation, and cellular damage. In addition, mRNA levels of apoptosis-associated speck-like CARD-domain protein (ASC), thioredoxin-interacting protein (TXNIP), NOD-like receptor family pyrin domain-containing 3 (NLRP3), and interleukin-1β (IL-1β) involved in the NLRP3 inflammasome accompanied by caspase-1 activity and IL-1β expression were significantly suppressed by 1,25(OH)2D in PA-treated hepatocytes. Moreover, upon PA exposure, 1,25(OH)2D-incubated AML-12 hepatocytes showed higher sirtulin 1 (SIRT1) expression and adenosine monophosphate-activated protein kinase (AMPK) phosphorylation. A SIRT1 inhibitor alleviated the beneficial effects of 1,25(OH)2D on PA-induced hepatic fat deposition, IL-1β expression, and caspase-1 activity. These results suggest that the favorable effects of 1,25(OH)2D on hepatic fat accumulation and inflammation may be, at least in part, associated with the SIRT1.

## 1. Introduction

At least 25–45% of the general adult population and 60% of patients with type 2 diabetes mellitus worldwide have non-alcoholic fatty liver disease (NAFLD) [1,2]. NAFLD is characterized by hepatic fat infiltration, which contributes to the excessive generation of reactive oxygen species (ROS) and oxidative stress and can progress to non-alcoholic steatohepatitis (NASH), cirrhosis, and hepatocellular carcinoma (HCC) [3,4,5]. Accumulating evidence indicates a strong relationship between low vitamin D levels and NAFLD prevalence [6,7,8]. Moreover, several clinical trials have reported that vitamin D supplementation reduces hepatic fat accumulation and inflammation in patients with NAFLD [9,10,11]. However, contradictory data regarding these positive effects have been reported [12,13]. Thus, the precise mechanism of action of vitamin D treatment in NAFLD needs to be investigated.

A series of recent studies have indicated a pivotal role of adenosine monophosphate-activated protein kinase (AMPK) and sirtuin 1 (SIRT1), a nicotinamide adenine dinucleotide (NAD)-dependent protein deacetylase, in the cellular and systematic regulation of lipid metabolism and energy homeostasis [14,15]. In addition to its roles in lipid and energy metabolism, there is a strong association with SIRT1 and hepatic lipid accumulation, oxidative stress, and inflammation. Liver-specific SIRT1 knockout mice fed a high-fat Western diet display impaired lipid metabolism and hepatic steatosis, inflammation, and endoplasmic reticulum stress [16]. Furthermore, the involvement of AMPK, regulated by SIRT1, in hepatic lipid disorders has been indicated by in vivo animal studies demonstrating that pharmacological or genetic modifications of AMPK activation are closely associated with hepatic lipid accumulation in rodents [17,18,19]. Taken together, the modulation of SIRT1/AMPK activation may be a potential therapeutic target for hepatic lipid accumulation and inflammation in NAFLD.

Accumulating evidence supports the involvement of fatty acid-induced inflammation in the activation of the NOD-like receptor family pyrin domain-containing 3 (NLRP3) inflammasome complex, comprising NOD-like receptor NLRP3, apoptosis-associated speck-like CARD-domain protein (ASC), and caspase-1 [20,21]. Moreover, NLRP3 inflammasome activation increases the release of interleukin-1β (IL-1β) which requires thioredoxin-interacting protein (TXNIP) [22]. Increased expression levels of NLRP3 and its components have been reported in both murine models and human subjects with liver disorders [21,23,24]. Additionally, NLPR3-specific deletion or pharmacological blockade ameliorates hepatic fat deposition, inflammation, and fibrosis and prevents the progression of NAFLD and other metabolic diseases [23,25]. Given the close association between the NLRP3 inflammasome and inflammatory stimuli, it may be a promising target for treating NAFLD-related inflammation and metabolic abnormalities. In addition, chemical and genetic modification of SIRT1 influence liver inflammation and fibrosis through the NLRP3 pathway [26,27,28]. Therefore, investigating the molecular mechanism of NLRP3-related liver inflammation mediated by SIRT1 has been paid attention.

This study investigated whether the most active metabolite of vitamin D, 1,25-dihydroxyvitamin D (1,25(OH)2D), suppresses the palmitic acid (PA)-induced increase in fat accumulation and inflammation, accompanied by reduced SIRT1/AMPK expression and NLRP3 inflammasome. Furthermore, whether the protective role of 1,25(OH)2D in hepatic fat deposition and inflammation is related to the NLRP3 inflammasome was determined in this current study.

## 2. Results

### 2.1. Influence of 1,25(OH)2D Treatment on Cell Viability in Murine AML-12 Hepatocytes

**Figure 1:**
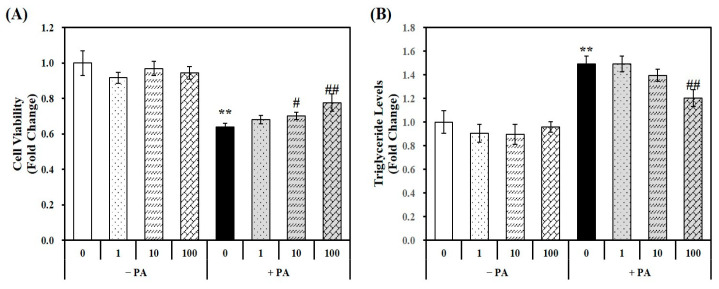
Effects of 1,25-dihydroxyvitamin D (1,25(OH)2D) on cell viability and lipid accumulation. Murine AML-12 hepatocytes were co-treated with palmitic acid (PA, 0.5 mM, 24 h) together with 1,25(OH)2D (0, 1, 10, or 100 nM for 24 h). The conversion to blue formazan (**A**) and intracellular triglyceride levels (**B**) were expressed as fold-change compared to vehicle control. The results are expressed as mean ± standard error of the mean. Experiments represent at least two or three independent experiments (*n* = 6–9 per group). ** *p* < 0.01 compared to vehicle control. # *p* < 0.05; ## *p* < 0.01 compared to PA control.

Using a PA-treated murine AML-12 hepatocyte cell line, the cellular cytotoxicity of 1,25(OH)2D and PA was analyzed using a 3-(4,5-dimethylthiazol-2-yl)-2,5-diphenyltetra-zolium bromide (MTT) assay. The MTT colorimetric assay measures the conversion of a tetrazolium dye to formazan by mitochondrial succinic dehydrogenases [29]. The results indicated that 1,25(OH)2D has no effect on cell viability without PA treatment. However, 24 h PA incubation significantly decreased hepatocytes by 36%, which was reversed by 1,25(OH)2D treatment in a dose-dependent manner, starting at a dose of 10 nM. Moreover, a 1.21-fold maximal increase was observed in hepatocytes treated with 100 nM 1,25(OH)2D-treated hepatocytes, compared to PA-treated cells (PA control, PA) (Figure 1A). 

### 2.2. Effects of 1,25(OH)2D on Fat Accumulation in PA-Treated AML-12 Mouse Hepatocytes

Murine AML-12 hepatocytes were incubated by PA (0.5 mM, 24 h) to induce hepatic fat accumulation. As shown in Figure 1B, a 1.49-fold increase in triglyceride (TG) levels was observed in PA-treated hepatocytes compared to vehicle control cells illustrating that the 24 h PA load induced fat deposition in hepatocytes. The 1,25(OH)2D treatment significantly attenuated PA-increased TG levels in a dose-dependent manner. A statistically significant 19.3% reduction was observed at a dose of 100 nM 1,25(OH)2D, indicating the protective effect of vitamin D on lipid accumulation in hepatocyte. 

### 2.3. Effects of 1,25(OH)2D on Oxidative Stress, Lipid Peroxidation, and Cellular Damage in PA-Treated AML-12 Mouse Hepatocytes

**Figure 2:**
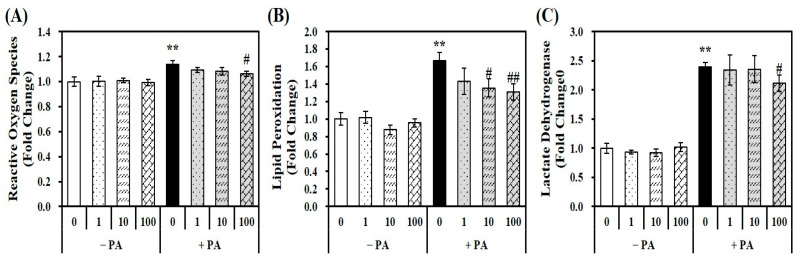
Influence of 1,25-dihydroxyvitamin D (1,25(OH)2D) on oxidative stress, lipid peroxidation, and intracellular damage. Murine AML-12 hepatocytes were co-treated with palmitic acid (PA, 0.5 mM for 24 h) together with 1,25(OH)2D (0, 1, 10, or 100 nM for 24 h). Reactive oxygen species (**A**), lipid peroxidation (**B**), and lactate dehydrogenase (**C**) were measured and expressed as fold-change compared to vehicle control. The results are expressed as mean ± standard error of the mean. Experiments represent at least two or three independent experiments (*n* = 6–9 per group). ** *p* < 0.01 compared to the vehicle control. # *p* < 0.05; ## *p* < 0.01 compared to PA control.

Given the close relationship between fat overload and increased oxidative stress, a fluorescent dichlorofluorescein assay with 2′,7′-dichlorofluorescin diacetate (DEFCA) was employed to measure the levels of oxidative stress [30]. PA loading (0.5 mM for 24 h) in hepatocytes significantly increased ROS by 1.13-fold compared to the vehicle control, which was suppressed by 1,25(OH)2D treatment (Figure 2A). To examine the influence of 1,25(OH)2D on PA-induced lipid peroxidation and intracellular damage in AML-12 cells, the intracellular levels of malondialdehyde (MDA) and lactate dehydrogenase (LDH) were measured. As shown in Figure 2B, PA significantly increased lipid oxidation by 1.66-fold, as measured by the MDA contents in hepatocytes, which were inhibited by 1,25(OH)2D in a dose-dependent manner. A statistically significant reduction in MDA levels by 1,25(OH)2D treatment started at 19% at a dose of 10 nM compared with PA-incubated hepatocytes (*p* < 0.05). In addition, PA-induced intracellular damage, as evidenced by a 2.5-fold increase in LDH concentrations, was dose-dependently inhibited by 1,25(OH)2D treatment, with a significant decrease observed at 100 nM (*p* < 0.05) (Figure 2C). These results suggest that 1,25(OH)2D improves PA-induced ROS production, lipid peroxidation, and cellular damage in AML-12 mouse hepatocytes.

### 2.4. Influence of 1,25(OH)2D on mRNA Expression Related to NLRP3 Inflammasome Components, IL-1β Contents, and Caspase-1 Activity in PA-Treated Hepatocytes

**Figure 3:**
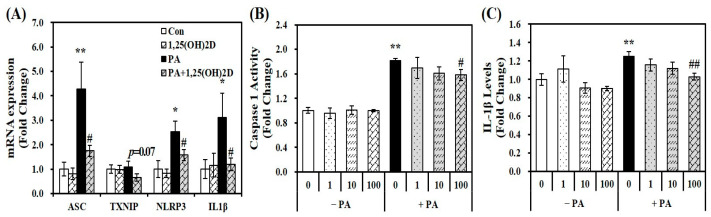
Effects of 1,25-dihydroxyvitamin D (1,25(OH)2D) on gene expression involved in NLRP3 inflammasome components (**A**), caspase-1 activity (**B**), and IL-1β contents (**C**) in palmitic acid (PA)-treated murine AML-12 hepatocytes. mRNA levels were determined by RT-PCR, normalized for all samples to β-actin, and presented as fold-change compared to vehicle control (Con). Caspase-1 activity and IL-1β levels were measured using commercial colorimetric ELISA kits, normalized to their respective protein levels, and expressed as fold-change compared to vehicle control (Con). Values are presented as mean ± standard error of the mean. Experiments represent two independent experiments (*n* = 8 per group). * *p* < 0.05, ** *p* < 0.01 compared to vehicle control (Con). # *p* < 0.05, ## *p* < 0.01 compared to PA control (PA). ASC, apoptosis-associated speck-like protein containing a CARD; IL, interleukin; NLRP3, NOD-, LRR- and pyrin domain-containing protein 3; TXNIP, thioredoxin interacting protein.

Based on emerging evidence that fatty acid-induced inflammation is involved in the activation of the NLRP3 inflammasome complex [20,21], gene expression of ASC, NLRP3, TXNIP, and IL-1β, involved in NLRP3 inflammasome components, intracellular IL-1β levels, and caspase-1 activity were analyzed to elucidate whether the protective effect of vitamin D on fat accumulation and inflammation might be associated with the NLRP3 inflammasome. Figure 3A demonstrates the effects of 1,25(OH)2D on gene expression of NLRP3 inflammasome compartments in PA-loaded AML-12 hepatocytes. 24 h PA incubation increased mRNA expression of ASC, TXNIP, NLRP3, IL-1β by 1.75-, 1.10-, 2.54-, and 3.11-fold, respectively. Furthermore, PA-increased ASC, TXNIP, NLRP3, and IL-1β mRNA levels were significantly downregulated by 59.1, 40.2, 37.9, and 61.8%, respectively by 1,25(OH)2D treatment (24 h, 100 nM). In addition, 24 h incubation of 1,25(OH)2D significantly inhibited PA-induced intracellular caspase-1 activity and IL-1β levels in a dose-responsive manner. Minimal caspase-1 activity and IL-1β levels were significantly reduced by 13% and 18%, respectively, at a dose of 100 nM 1,25(OH)2D, compared to PA-treated cells (*p* < 0.05) (Figure 3B,C). These results illustrate the favorable effect of vitamin D on the NLRP3 pathway in hepatocytes treated with PA.

### 2.5. Effects of 1,25(OH)2D on AMPK Phosphorylation and SIRT1 Protein Abundance in PA-Treated AML-12 Mouse Hepatocytes

**Figure 4:**
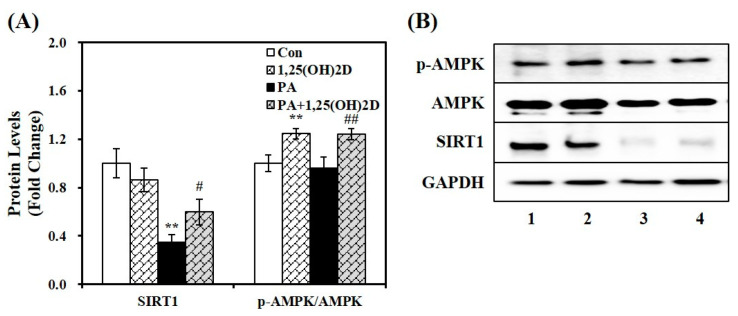
Effects of 1,25(OH)2D on AMPK phosphorylation and SIRT1 protein abundance in palmitic acid (PA)-treated hepatocytes. AML-12 mouse hepatocytes were treated with 0.5 mM PA (lanes 3 and 4) together with 100 nM 1,25(OH)2D (lanes 2 and 4) for 24 h. The density of the signal was quantified, normalized by GAPDH or AMPK, and expressed as fold-change compared to vehicle control (Con, lane 1) (**A**). Representative Western blots for p-AMPK, AMPK, SIRT1, and GAPDH (**B**). Experiments were repeated at least twice (*n* = 4–6 per group). Values are presented as mean ± standard error of the mean. Bars with asterisks indicate significant differences compared to vehicle control (Con) or PA control (PA). ** *p* < 0.01 compared to vehicle control (Con, lane 1). # *p* < 0.05, ## *p* < 0.01 compared to PA control (PA, lane 3). AMPK, adenosine monophosphate-activated protein kinase; GAPDH, glyceraldehyde-3-phosphate dehydrogenase; SIRT1, sirtuin1.

Regarding the role of SIRT1 in hepatic lipid metabolism, SIRT1 protects against lipid-overloaded NAFLD via AMPK activation [31,32]. Western blotting with antibodies against SIRT1, AMPK phosphorylation (p-AMPK), and AMPK was performed to investigate the beneficial effects of vitamin D on hepatic fat deposition and inflammation by modulating SIRT1/AMPK signaling. As shown in Figure 4A,B, PA influx (lane 3) decreased SIRT1 protein expression and AMPK phosphorylation by 65% and 4%, respectively, compared to vehicle control (lane 1). Additionally, 24 h incubation with 1,25(OH)2D significantly upregulated PA-decreased SIRT1 levels and AMPK phosphorylation (lane 4) by 1.72- and 1.30-fold, respectively, when compared to PA controls (lane 3). This suggests that vitamin D-reduced fat accumulation and inflammation in NAFLD might be associated with the SIRT1 pathway. 

### 2.6. Influence of a SIRT1 Inhibitor on 1,25(OH)2D-Decreased TG Deposition and Inflammation in PA-Treated Hepatocytes

**Figure 5:**
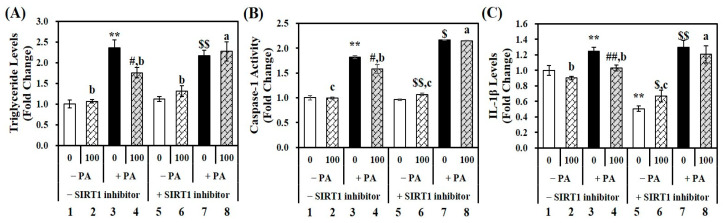
Effects of 1,25(OH)2D on fat deposition and inflammation through SIRT1 in palmitic acid (PA)-treated AML-12 mouse hepatocytes. AML-12 cells were incubated with PA (0.5 mM. 24 h; lanes 3, 4, 7, and 8) to induce hepatic steatosis. Hepatocytes were co-treated with 1,25(OH)2D (100 nM, 24 h; lanes 2, 4, 6, and 8), combined with EX-527 (10 μM, 24 h; lanes 5, 6, 7, and 8). (**A**) Triglyceride concentration, (**B**) caspase-1 activity, and (**C**) IL-1β levels were measured using commercial colorimetric ELISA kits, normalized to their respective protein levels, and expressed as fold-change compared to vehicle control in the absence of SIRT1 inhibitor (lane 1). Values are expressed as means ± standard error of the mean (*n* = 6) of two independent experiments. ** *p* < 0.01 compared to vehicle control in the absence of EX-527 (lane 1). # *p* < 0.05, ## *p* < 0.01 compared to PA control in the absence of EX-527 (lane 3). $ *p* < 0.05, $$ *p* < 0.01 compared to vehicle control in the presence of EX-527 (lane 5). Different letters (a, b, c) indicate the statistically different effects of 1,25(OH)2D, determined by one-way analysis of variance (ANOVA), followed by Student–Newman–Keuls multiple comparison post hoc test.

To investigate whether the inhibitory effect of 1,25(OH)2D on TG deposition and inflammation is related to the SIRT1-NLRP3 pathway, a specific SIRT1 inhibitor, EX527 [33,34] was employed in PA-treated hepatocytes. As shown in Figure 5A, in the absence of SIRT1 inhibitor, 0.5 mM PA exposure (lane 3) significantly increased TG contents by 2.36-fold in hepatocytes compared to vehicle control cells (lane 1). This PA-induced TG accumulation was significantly reduced by 100 nM 1,25(OH)2D by 25.5% (lane 4), compared to the PA-treated cells (lane 3) in the absence of the SIRT1 inhibitor. Interestingly, usage of the SIRT1 inhibitor abolished vitamin D-decreased fat deposition in PA-overloaded hepatocytes (lane 7 and 8). Thus, SIRT1 inhibition in AML-12 hepatocytes affected PA-induced fat accumulation. 

The activation of caspase-1 and pro-inflammatory IL-1β levels were measured in the absence and presence of SIRT1 inhibitor to investigate the link between SIRT1 and hepatic inflammation relating to NLRP3 inflammasome activation. Figure 5B illustrates effect of SIRT1 on PA-decreased caspase-1 activity. A statistically significant 1.82-fold increase was observed in PA-treated cells (lane 3) compared to vehicle control cells in the absence of SIRT1 inhibitor (lane 1). Without a specific SIRT1 inhibitor, 1,25(OH)2D treatment (lane 4) significantly inhibited PA-induced caspase-1 activation, compared to PA control cells (lane 3). This significant reduction was decreased by 0.6% when comparing between the PA (lane 7) and the PA+1,25(OH)2D (lane 8) in the presence of SIRT1 inhibitor. Additionally, hepatic IL-1β concentration was significantly increased by 1.25-fold by PA incubation (lane 3) compared vehicle control (lane 1). As shown in Figure 5C, 24 h incubation of 100 nM 1,25(OH)2D to PA-loaded hepatocytes in the absence of SIRT1 inhibitor significantly decreased hepatic IL-1β levels by 17.6% (lane 3), compared to PA control (lane 4). Using a selective SIRT1 inhibitor alleviated 1,25(OH)2D-decreased IL-1β contents by 7.1% compared to PA-treated cells with EX-527 (lane 7 vs. 8). 

In summary, the statistically significant reduction in hepatic TG levels, caspase-1 activity, and IL-1β concentrations induced by 1,25(OH)2D incubation in PA-treated cells without EX-527 were diminished by incubation with a SIRT1 inhibitor, EX-527 (Figure 5A–C). There was no statistical difference in intracellular TG levels, IL-1β contents, and caspase-1 activity between PA-treated cells and hepatocytes with PA+1,25(OH)2D in the presence of SIRT1 inhibitor. Thus, 1,25(OH)2D-induced changes in PA-loaded fat accumulation and inflammation may be involved in SIRT1 expression in murine AML-12 hepatocytes. 

## 3. Discussion

Numerous clinical and epidemiological studies have reported a strong relationship between low vitamin D levels and NAFLD. The present study demonstrates that 1,25(OH)2D treatment reduced fat accumulation, IL-1β expression, and caspase-1 activity in PA-overloaded AML-12 hepatocytes. This study is the first to show that 1,25(OH)2D treatment significantly increased hepatic SIRT1 protein and AMPK phosphorylation expression and reduced mRNA levels involved in the NLRP3 inflammasome compartment along with a decrease in IL-1β levels and caspase-1 activity. Furthermore, a selective inhibitor of the SIRT1 counteracted the positive effects of 1,25(OH)2D on hepatic steatosis and inflammation. These results suggest that vitamin D may decrease intracellular fat accumulation and inflammation concurrently with a decrease in SIRT1 expression.

Liver diseases, ranging from exacerbated hepatic fat deposition to hepatic inflammation, ballooning, injury, fibrosis, and cellular death, have been recognized as hallmarks of NAFLD, all of which contribute to the progression to NASH, cirrhosis, HCC, and death [3,4,5]. Thus, prevention and treatment of steatosis and inflammation have attracted attention as potential therapeutic targets. Several observational clinical studies have shown an inverse association between vitamin D status and NAFLD prevalence; however, there is no clear consensus regarding the effects of vitamin D supplementation on NAFLD [6,7,12,13]. Here, we evaluated whether 1,25(OH)2D supplementation modulated hepatic fat accumulation in PA-treated AML-12 hepatocytes. A significant reduction in TG levels was observed in hepatocytes treated with 100 nM 1,25(OH)2D compared to vehicle-treated cells. Consistent with the data presented here, supplementation with cholecalciferol or 1,25(OH)2D reduces TG accumulation in the livers of obese C57BL/6J mice fed a high-fat diet with drinking water containing 10% sucrose, or in diabetic SD rats fed a high-fat and high-sugar diet [35,36]. 

Oxidative stress results from an imbalance between ROS production and the scavenging capacity. Lipid accumulation in the liver induces lipid peroxidation, ROS toxicity, and cell death, leading to the progression from simple steatosis to NASH [3,4]. Patients with NAFLD have higher hepatic MDA and ROS levels [37]. In the present study, 1,25(OH)2 treatment significantly reduced PA-induced ROS levels, lipid peroxidation (measured by MDA levels), and cellular damage (indicated by LDH contents) in hepatocytes. The mechanism by which vitamin D treatment improves NAFLD may be associated with a decrease in serum MDA levels in response to increased vitamin D status. In a randomized, placebo-controlled, double-blind study, a high oral dose of vitamin D lowered serum MDA levels and increased antioxidant capacity in patients with NAFLD [38]. Similarly, vitamin D ameliorated oxidative stress and inflammation in both patients with NAFLD and rat livers treated with carcinogens [39,40]. Thus, therapeutic interventions involving vitamin D for NAFLD-related oxidative stress may be helpful in preventing the progression to NASH.

When investigating important protective roles against hepatic fat metabolism and inflammation, several studies have identified two key nutrient sensors, SIRT1 and AMPK. For example, hepatic-specific deletion of SIRT1 in mice fed with a normal control diet resulted in severe lipid deposition [41]. Moreover, liver-specific SIRT1 knockout mice fed a high-fat Western diet display impaired lipid metabolism and develop hepatic steatosis, inflammation, and endoplasmic reticulum stress [16]. In contrast, hepatic SIRT1 overexpression in mice protects against high fat diet-induced hepatic steatosis [42]. Furthermore, SIRT1 ameliorates hepatic steatosis through the activation [31,43]. Low AMPK activity has been observed in fatty livers of rodents [44]. AMPK activators significantly reduce liver fat levels, supporting the potential role of AMPK activation in fatty liver [18,43,44,45]. In the current study, PA incubation significantly decreased SIRT1 protein expression and AMPK activation, as calculated by dividing p-AMPK by AMPK expression, which was significantly reversed by 1,25(OH)2D treatment (100 nM for 24 h) in AML-12 hepatocytes. Thus, the inhibitory effect of 1,25(OH)2D on fat accumulation may be associated with increased SIRT1 expression and AMPK phosphorylation in hepatocytes. 

Upon activation by fat overload, the NLRP3 inflammasome complex is assembled and related to the recruitment of the ASC protein, which in turn oligomerizes with pro-caspase-1 [20,21,22], leading to the activation of caspase-1 and the secretion of pro-inflammatory IL-1β and IL-18 [20]. Given that fat overload can activate the NLRP3 inflammasome, the role of the NLRP3 inflammasome in the pathogenesis and development of NAFLD needs to be elucidated. Increased influx of saturated fatty acids in the liver activates the NLRP3 inflammasome and IL-1β secretion, which is involved in ROS generation [21,46]. Genetic modifications or pharmacological inhibitors of the NLRP3 inflammasome alleviate HFD-induced hepatic steatosis, hepatocyte inflammation, and fibrogenesis [47,48]. In contrast, hepatic-specific or global overexpression of NLRP3 results in inflammation and fibrosis, which in turn leads to the progression to NASH [49,50]. Here the present study demonstrated that mRNA levels of ASC, NLRP3, TXNIP, and IL-1β related to NLRP3 inflammasome compartments were significantly inhibited by 1,25(OH)2D treatment (100 nM, 24 h) in PA-overloaded hepatocytes. Therefore, 1,25(OH)2D-decreased hepatic inflammation might be associated with NLRP3 inflammasome activation in PA-loaded AML-12 mouse hepatocytes. 

Accumulating evidence indicates that the NLRP3 inflammasome signaling pathway is involved in the progression of hepatic steatosis to NASH through SIRT1. The beneficial effects of resveratrol, a SIRT1 activator on hepatic fat accumulation and inflammation are associated to inhibition of NLRP3 inflammasome pathway, which ultimately plays a pivotal role in preventing liver injury [26]. In carbon tetrachloride (CCl4)-induced hepatic fibrosis rat models, SIRT1 overexpression attenuates the conversion of macrophages to the pro-inflammatory M1 type and inhibits the release of inflammatory cytokines via downregulation of the nuclear factor kappa B (NF-κB)/NLRP3 pathway [28]. In liver-specific SIRT1 knockout mice, prolonged inhibition of SIRT1 contributes to persistent activation of NLRP3 and produces the pro-inflammatory cytokine, IL-1β, which aggravates severe liver injury, fibrosis, and inflammation [51]. In the present study, EX-527 which binds to the nicotinamide binding pocket of SIRT1 required NAD^+^ was employed to investigate whether the inhibitory effect of 1,25(OH)2D treatment on hepatic NLRP3 inflammation activation is involved in SIRT1. Indeed, 1,25(OH)2D significantly down-regulated PA-increased IL-1β expression, caspase-1 activity, and TG deposition which were abolished by a selective SIRT1 inhibitor, EX-527. These findings suggest that SIRT1 might serve as a molecular therapeutic target for 1,25(OH)2D via the NLRP3 inflammasome inhibition in NAFLD. However, it has not been elucidated whether modulation of the SIRT1-NLRP3 inflammasome pathway by 1,25(OH)2D, which influences hepatic fat deposition and inflammation, is related to AMPK activity, an important downstream of SIRT1. Increasing evidence suggests that a known AMPK activator, metformin, suppresses macrophage IL-1β production and increases TXNIP phosphorylation and degradation [52,53]. Therefore, a following study is needed to determine by which the SIRT1-NLRP3 inflammasome pathway that affects the favorable effects of 1,25(OH)2D on hepatic fat accumulation and inflammation might be associated with AMPK activity using AMPK activators such as metformin and genetic modification. 

In conclusion, the present study suggests that 1,25(OH)2D treatment protects against PA-induced hepatic TG accumulation, lipid peroxidation, and cell death, suppresses the NLRP3 inflammasome, and increases SIRT1 and AMPK phosphorylation. The favorable effect of 1,25(OH)2D on hepatic steatosis and inflammation, at least in part through SIRT1 in AML-12 mouse hepatocytes. Taken together, 1,25(OH)2D may serve as a novel therapeutic agent for PA-induced hepatic steatosis.

## 4. Materials and Methods

### 4.1. Cell Culture and Treatment

Murine AML-12 hepatocytes obtained from the American Type Culture Collection (ATCC, CRL-2254; Manassas, VA, USA) were cultured in DMEM/F-12 (Gibco, Grand Island, NY, USA) supplemented with 10% fetal bovine serum (Gibco), 100 U/mL of penicillin and 100 μg/mL of streptomycin (Gibco), 0.1 µM dexamethasone, and a mixture of insulin, transferrin, and selenium (Sigma-Aldrich, St. Louis, MO, USA) at 37 °C in 95% air and 5% CO_2_ atmosphere. AML-12 hepatocytes were treated with PA (0.5 mM, 24 h; Sigma) prepared as previously described [54], together with vehicle control (0.1% ethanol, Sigma) or 1,25(OH)2D (Sigma) dissolved in absolute ethanol at the given concentrations. 

### 4.2. Cell Viability 

After 24 h of 1,25(OH)2D treatment at the desired concentration, MTT solution (5 mg/mL, Sigma) was added to PA-treated murine AML-12 hepatocytes for 1 h, followed by dissolution in DMSO (Sigma). A Varioskan plate reader (Thermo Scientific, Waltham, MA, USA) was used to measure the absorbance at 570 nm. Cell viability was represented as fold-change compared to vehicle-treated cells without PA or 1,25(OH)2D treatment.

### 4.3. Triglyceride Levels

To prepare cells for the assay, 5% Nonidet P-40 (NP-40) substitute (Sigma) was used on harvested AML-12 hepatocytes for homogenization. The homogenate was then heated at 80–100 °C followed by cooling at room temperature. As described previously [54], intracellular TG levels were quantified using a commercial TG assay kit (Abcam, Cambridge, MA, USA) and normalized to their respective protein amounts measured with a bicinchoninic acid (BCA) protein assay kit (Thermo Scientific). Values are expressed as fold-change compared to the vehicle control. 

### 4.4. Reactive Oxygen Species (ROS) Measurement

ROS levels were measured using DCFDA cellular ROS detection kit (Abcam) according to previously described methods [55]. Briefly, AML-12 hepatocytes were incubated with PA (0.5 mM) together with 1,25(OH)2D at various concentrations. After 24 h incubation, hepatocytes were washed with phosphate-buffered saline (PBS, pH 7.4, Gibco), incubated with 20 μM DCFHDA in serum-free medium for 45 min at 37 °C. Cellular ROS levels were determined at 485 nm/535 nm (excitation/emission) and expressed as fold-changes compared to the vehicle control.

### 4.5. Malondialdehyde (MDA) Measurement 

A commercial colorimetric thiobarbituric acid reactive substances assay kit (Abcam) was used to measure the MDA levels. Based on the reactivity of thiobarbituric acid with MDA, an end product of lipid peroxidation, a red adduct was generated and quantified at an absorbance of 532 nm. Lipid peroxidation was normalized to their respective protein concentrations, as determined using a BCA protein assay kit (Thermo Scientific) and represented as a fold-change compared to the vehicle control. 

### 4.6. Determination of Lactate Dehydrogenase (LDH) Levels 

To evaluate the effect of 1,25(OH)2D on cell damage in PA-treated AML-12 hepatocytes, LDH levels were measured using a commercially available colorimetric kit (Abcam). LDH, which reduces NAD to NADH and reacts with a specific probe to produce a colored product at 450 nm. LDH levels were normalized to their respective protein levels and expressed as fold-change compared to the vehicle control. 

### 4.7. Measurement of Caspoase-1 Activity 

The effect of 1,25(OH)2D treatment on caspase-1 activity was measured using a commercial fluorometric assay kit (Abcam) based on the cleavage of 7-amino-4-trifluoromethyl coumarin (AFC). A yellow-green fluorescence was produced upon AFC cleavage by caspase-1 during 1–2 h incubation at 37 °C and quantified on a fluorescence plate reader (Thermo Scientific) at 400 nm/505 nm (excitation/emission), normalized by their respective protein amounts, and expressed as fold-change compared to the vehicle control. 

### 4.8. Determination of Interleukin-1β (IL-1β) Levels

Intracellular IL-1β protein levels were quantified using a commercial ELISA kit (Abcam) including IL-1β capture and detection antibodies, according to the manufacturer’s protocol. Briefly, 1,25(OH)2D-treated hepatocytes were incubated with an antibody cocktail for 1 h at room temperature, washed, and exposed to 3,3′,5,5′-tetramethylbenzidine (TMB) for 10 min. TMB is a peroxidase substrate that changes color during reactions. After adding the stop solution, the absorbance was measured at 450 nm using a Varioskan plate reader (Thermo Scientific). 

### 4.9. RNA Isolation, Reverse Transcription, and Quantitative Real-Time Polymerase Chain Reaction (qRT-PCR)

Total RNA was extracted from murine AML-12 hepatocytes using the RNeasy Mini Kit (Qiagen, Valencia, CA, USA) following the manufacturer’s instructions. cDNAs were generated from 1 μg RNA using an MMLV Reverse Transcriptase Kit (Bioneer, Daejeon, Republic of Korea) and GeneAMP^®^ PCR system 2700 (Applied Biosystems, Foster City, CA, USA) with an incubation at 37 °C for 60 min followed by 5 min at 95 °C. Individual reactions for target and β-actin were carried out using qRT-PCR (Rotor-Gene Q thermocycler, Qiagen, Hilden, Germany) as follows: 95 °C for 10 min, followed by 40 cycles of denaturation (95 °C for 15 s), annealing (60 °C for 20 s), and extension (72 °C for 20 s). The relative expression of each target was calculated using the comparative Ct (ΔΔCT) method [56], normalized to that of β-actin, and presented as fold-change compared to vehicle controls. The primer sequences are listed in Table 1. 

### 4.10. Western Blot Analysis

AML-12 hepatocytes were washed in ice-cold PBS (Gibco) and lysed in cold RIPA lysis buffer (Sigma) containing protease and phosphatase inhibitors (Roche, Indianapolis, IN, USA) via homogenization. Protein levels were quantified using a BCA protein assay kit (Thermo Scientific). Equal amounts of 10 μg protein samples were loaded and separated with a 4–15% gradient SDS polyacrylamide gel (Bio-Rad Laboratories, Inc., Hercules, CA, USA) and transferred to polyvinylidene difluoride (PVDF) membranes (Bio-Rad). The membranes were blocked with 5% bovine serum albumin (BSA)-containing Tris-Buffered saline with Tween-20 (Bio-Rad) for 1 h at room temperature, and then probed with the following primary antibodies overnight at 4 °C: p-AMPK (2531), AMPK (2532), SIRT1 (9475), and GAPDH (5174) (Cell Signaling Technology, Danvers, MA, USA). The secondary antibody, HRP-conjugated anti-rabbit IgG (Cell Signaling) was incubated for 1 h at room temperature. The immunoreactive bands were developed with the Westar Sun (Cyanagen, Bologna, Italy) and Azure 300 Imaging Systems (Azure Biosystems, Dublin, CA, USA). Densitometry analysis was performed using ImageJ software (64-bit Java 1.8.0_172; National Institutes of Health, Bethesda, MD, USA).

### 4.11. Statistical Analyses

SPSS software (version 28; IBM Corporation, Armonk, NY, USA) was used for statistical analyses. All results are presented as mean ± standard error of the mean (SEM). One-tailed Student’s *t*-tests were performed used to identify the statistical differences. Statistical significance was defined as *p* < 0.05.

## Figures and Tables

**Table 1:** Primers used for RT-qPCR.

Gene	Accession Number	Primer Sequences (5′-3′)
ASC	NM_023258.4	F: TTA ATC CCA GCA ACC AGG AG R: CTT GAG TTA GGC CAG CCT TG
β-actin	NM_007393	F: GGA CCT GAC AGA CTA CCT CA R: GTT GCC AAT AGT GAT GAC CT
IL-1β	NM_008361.4	F: GCC CAT CCT CTG TGA CTC AT R: AGG CCA CAG GTA TTT TGT CG
NLRP3	NM_145827.3	F: ATG CTG CTT CGA CAT CTC CT R: AAC CAA TGC GAG ATC CTG AC
TXNIP	NM_023719.2	F: GGA GCC TGG GTG ACA TTC TA R: TGC ACA GTT CTC AGG TGG AG

ASC, apoptosis-associated speck-like protein containing a CARD; IL, interleukin; NLRP3, NOD-, LRR- and pyrin domain-containing protein 3; TXNIP, thioredoxin interacting protein.

## Data Availability

Data are contained within the article.
